# Theoretical Analysis of Magnetic Coupling in the Ti_2_C Bare MXene

**DOI:** 10.1021/acs.jpcc.2c07609

**Published:** 2023-02-14

**Authors:** Néstor García-Romeral, Ángel Morales-García, Francesc Viñes, Ibério de P. R. Moreira, Francesc Illas

**Affiliations:** Departament de Ciència de Materials i Química Física & Institut de Química Teòrica i Computacional (IQTCUB), Universitat de Barcelona, c/ Martí i Franquès 1-11, 08028 Barcelona, Spain

## Abstract

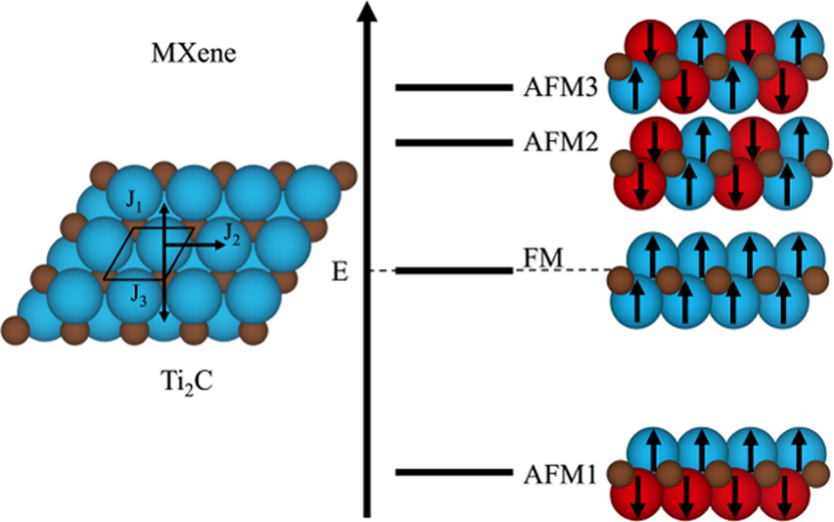

The nature of the electronic ground state of the Ti_2_C MXene is unambiguously determined by making use of density functional theory-based calculations including hybrid functionals together with a stringent computational setup providing numerically converged results up to 1 meV. All the explored density functionals (i.e., PBE, PBE0, and HSE06) consistently predict that the Ti_2_C MXene has a magnetic ground state corresponding to antiferromagnetic (AFM)-coupled ferromagnetic (FM) layers. A spin model, with one unpaired electron per Ti center, consistent with the nature of the chemical bond emerging from the calculations, is presented in which the relevant magnetic coupling constants are extracted from total energy differences of the involved magnetic solutions using an appropriate mapping approach. The use of different density functionals enables us to define a realistic range for the magnitude of each of the magnetic coupling constants. The intralayer FM interaction is the dominant term, but the other two AFM interlayer couplings are noticeable and cannot be neglected. Thus, the spin model cannot be reduced to include nearest-neighbor interactions only. The Néel temperature is roughly estimated to be in the 220 ± 30 K, suggesting that this material can be used in practical applications in spintronics and related fields.

## Introduction

Since the discovery and isolation of graphene in 2004,^1^ the family of two-dimensional (2D) materials has been continuously growing and gaining interest and importance in various applications related to materials science.^2^ The interest in these materials comes from their exceptional properties with implications in a large number of fields, including electronic devices, optoelectronics, catalysis, energy storage, sensing platforms, and solar cells.^2^ However, it has been well demonstrated that the most extensively studied 2D materials, such as C-based graphene,^1^ graphynes,^3^ and grazynes^4^ but also boron nitride,^5^ black phosphorus,^6^ and silicene,^7^ are generally less stable, and most of them are not suitable for spintronics applications due to their general lack of intrinsic magnetism which calls for alternatives.

In 2011, a new family of low-dimensional materials was discovered that involves 2D transition-metal carbides, nitrides, and carbonitrides. These new materials are commonly referred to as MXenes.^8,9^ They can be synthesized by selective chemical etching of A elements in the corresponding atomic layers of layered three-dimensional materials known as MAX phases. These are identified with a M_*n*+1_AX_*n*_ general formula with M = early transition metal, A = element of group XIII or XIV, X = C and/or N; *n* = 1–3 which determines the MXene thickness.^10,11^ Conventional MXenes have a general formula M_*n*+1_X_*n*_T_*x*_ where T_*x*_ stands for chemical groups bonded to MXenes surfaces as a consequence of synthetic conditions, the most common ones being OH, H, O, or F.^12,13^ Although these can be efficiently removed through newly reported protocols^14,15^ allowing us to generate pristine MXene surfaces with general formula M_*n*+1_X_*n*_.^12,14,16^

Due to their large diversity of composition, MXenes have a wide range of properties and applications depending on their constituent elements, thickness and/or surface terminations, also referred to as functionalization.^9^ Among the different applications of MXenes, one can easily highlight water dissociation^17^ and purification,^18^ CO_2_ abatement,^19^ electrochemical capacitors, and their use in alkali-ion batteries,^15,20−22^ lubrication, gas- and bio-sensors, and thermo- and electro- and photo-catalysis.^16,20,21,23−25^ There is, of course, interest in examining the applications related to their possible magnetic properties. This implies finding whether their electronic ground state has a magnetic character as well as understanding their related magnetic features for possible applications in spintronics. In fact, a considerable research effort has been addressed to investigate the magnetic properties of different types of MXenes such as *i*-MXenes,^26−28^*o*-MXenes,^26,29−34^ Janus MXenes,^35,36^ functionalized MXenes,^37−44^ and pristine MXenes.^39^ In addition, there are studies inducing magnetism in non-magnetic (NM) MXenes via external inputs, for example, mechanical strain,^15,45,46^ functionalization,^26,44,47^ external electric fields,^36,48^ vacant formation,^28,32^ or doping with organic molecules^49^ or atoms,^50^ among others. However, in spite of the large number of published studies, the literature evidences a noticeable disparity of results even for the simplest M_2_C MXene. It is very likely that, to a large extent, the results are biased by the use of pure GGA functionals that lead to metallic (or semimetallic Dirac-like) closed shell ground states.^51^

In the case of Ti_2_C, there is consensus that the electronic ground state is magnetic. However, there is a great diversity of results regarding the relative stability of the states that exhibit different spin ordering, and the information in the literature is not complete or can lead to a misunderstanding. A previous study states that this MXene features a magnetic ground state but without specifying the magnetic order.^38^ Several articles report a ferromagnetic (FM) ground state,^45,46,52^ whereas others predict it to be antiferromagnetic (AFM).^42,48,53−57^ Nevertheless, one must point out that one of the four references reporting a FM ground state for Ti_2_C is a review,^38^ and two other did not consider other magnetic solutions.^45,52^ Part of this diversity can be attributed to the use of different theoretical strategies. All methods are based on density functional theory (DFT) but rely on different approximations to the unknown universal exchange–correlation density functional. The use of different basis sets and numerical settings also represents an additional problem when comparing the results from each study. Among the different methods used to describe the electronic structure of Ti_2_C in the literature, we mention the generalized gradient approximation (GGA)-based functional proposed by Perdew–Burke–Ernzerhof^58^ (PBE), either without or with Hubbard correction (PBE + *U*), the GGA approach by Perdew–Wang^59^ (PW91) which, at least for bulk transition metals, behaves very similar to PBE,^60,61^ and the short-range corrected hybrid Heyd–Scuseria–Ernzerhof^62^ (HSE06) density functional. All these functionals may or not include dispersion effects. The GGA and hybrid functionals belong to two families of DFT-based methods that are broadly used in computational materials science although with pros and cons. GGA functionals are very accurate for metals,^60,61^ but results tend to be system dependent and clearly fail to correctly predict the band gap of insulating magnetic materials as simple as NiO.^51^ On the contrary, hybrid functionals properly describe oxides and related materials but do not perform well enough for metals.^61^ In addition, finding the amount of non-local Fock exchange required to reproduce the experimental band gap remains an unsolved problem,^51,63^ and the same applies to the parameters defining the range separation in HSE06.^64^ The alternative PBE + *U* approximation provides a computational efficient scheme to improve the description of magnetic states, but it is difficult to be applied in complex systems with different types of localized electrons thus requiring with more than a *U* value. The choice of *U* is also a delicate issue, one can for instance choose a value that reproduces an experimental known property or can estimate it from macroscopic properties as the dielectric constant.^65^ Both requiring information that should be provided by the Hamiltonian system and not vice versa.

The lack of a common, sometimes sufficiently accurate, setup makes the comparison of the magnetic properties for the same MXene, Ti_2_C, further complicated and, at this point, the magnetic nature of the Ti_2_C ground state is uncertain. One of the goals of the present work it to definitively settle this issue by providing numerically accurate results regarding the relative energy stability of the possible NM and different magnetic configurations of pristine Ti_2_C. We will present compelling evidence that, regardless of the DFT method used, the electronic ground state of these MXenes is magnetic and that the lowest magnetic state exhibits an AFM spin ordering such that the Ti atoms within each metallic layer are ferromagnetically coupled with AFM coupling between the Ti layers; in agreement with some of the previous studies in the literature.^42,48,53−57^ In addition, a spin model Hamiltonian is also presented that is compatible with the nature of the chemical bond, thus allowing us to rationalize the obtained results with the magnetic couplings derived from total energy differences as previously done for other material including oxides and high critical temperature cuprate parent compounds.^66^

## MXene Models and Computational Details

The calculations presented in the present work have been obtained by means of the Vienna ab initio simulation package (VASP).^67^ Before describing in more detail the overall procedure used in this work, one must point out that, to definitively settle the discussion regarding the nature of the electronic ground state of the Ti_2_C MXenes, it is necessary to provide results numerically converged up to 1 meV per cell. To this end, a common and numerically very tight setup is selected and used to explore the magnetic properties of Ti_2_C MXene. In addition, three different density functionals are used to explore non-spin-polarized and spin-polarized solutions. For the latter, the FM and different AFM spin arrangements are also scrutinized. The functionals used are PBE,^58^ PBE0,^68,69^ and HSE06,^62^ the former is a GGA-based density functional, and the rest are hybrid functionals both including a 25% of Fock exchange with the latter also involving a range separation for the non-local exchange with a screening parameter ω = 0.2 Å^–1^.

**Figure 1:**
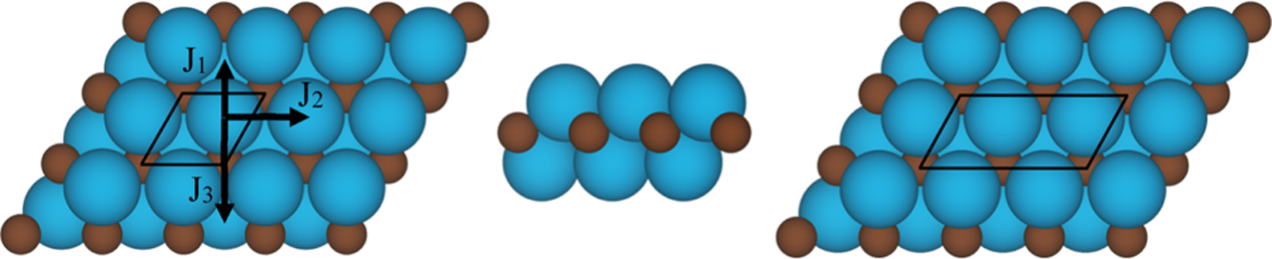
Top view of fully relaxed bare *p*(1 × 1) Ti_2_C MXene surface (left), side view of the Ti_2_C MXene (middle), and the top view of *p*(2 × 1) supercell used to compute the different spin-polarized solutions (right). Top view of the supercells and spin exchange paths for the magnetic coupling parameters, as defined in the text, are also displayed in *p*(1 × 1) cell top view.

A first set of calculations was carried out to optimize the lattice parameter using a *p*(1 × 1) unit cell, see Figure 1, left panel, using the PBE^58^ density functional neglecting or including spin polarization. In the latter case, several solutions were explored, as detailed below. Also, to properly represent this 2D material within a fully periodic approach, a 15 Å vacuum width is introduced along the direction perpendicular to the surface plane to avoid interaction between the 2D replicas. For this unit cell, the Kohn–Sham equations are solved in a plane-wave basis set with a 700 eV kinetic energy cut-off which is significantly larger than the recommend value and thus leads to higher numerical accuracy, ensuring that the error on energy differences used to compute the magnetic coupling constants (*vide infra*) is below 1 meV. The projector-augmented wave method is used to account for the interaction between the valence and the core electron densities.^70^ A very dense 13 × 13 × 1 Monkhorst–Pack grid of special *k*-points grid is used to carry out the numerical integrations in the reciprocal space, thus also ensuring numerical convergence to the chosen accuracy. To facilitate convergence of the self-consistent field procedure, all calculations were carried out using a smearing width of 0.01 eV for partial occupancies with the Methfessel–Paxton method, but all calculated energy values were then extrapolated to 0 K. A 10^–6^ eV threshold was chosen as electronic convergence criterion, again to ensure numerical convergence. The geometry optimizations were considered converged when the forces acting on the nuclei were all below 0.01 eV·Å^–1^.

**Figure 2:**
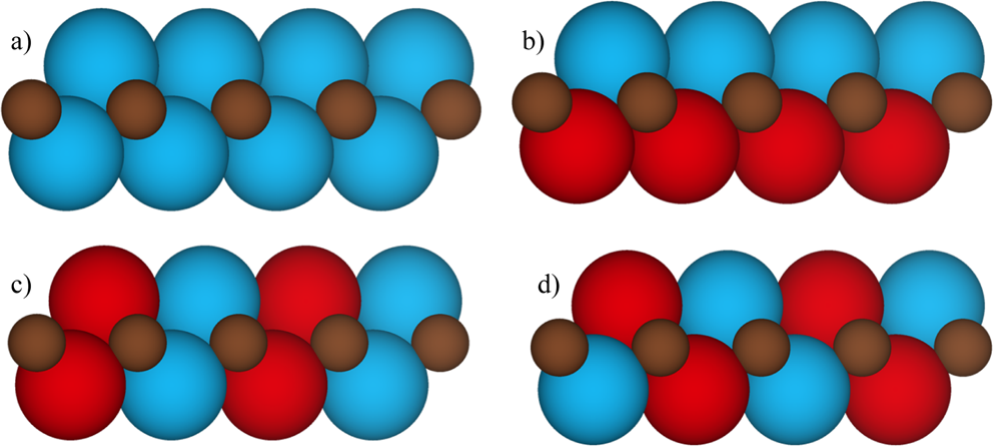
Schematic representation of the (a) FM, (b) AFM1, (c) AFM2, and (d) AFM3 magnetic solutions where Ti atoms with spin up and spin down are depicted in blue and red, respectively.

In subsequent calculations, carried out for each PBE-optimized structure corresponding to each of the different explored solutions, the total energy was evaluated for the different solutions using each of the above described functionals. For this *p*(1 × 1) unit cell, the possible solutions are the non-spin-polarized which, obviously, is NM, the FM with parallel spins mainly localized in the Ti atoms, and the AFM one (AFM1) with antiparallel spins in the Ti atoms. Additional spin orderings were explored using a *p*(2 × 1) supercell; these are the AFM2 and AFM3 described in detail in the next section. For consistency, the FM and AFM1 solutions were also obtained for the *p*(2 × 1) supercell, see Figure 2, a and b panels. From all the explored solutions, a clear picture of the electronic structure of this MXene is obtained. In the following, compelling evidence will be presented that this material exhibits an AFM1 ground state, and the different relevant magnetic solution and corresponding magnetic couplings are mapped into an appropriate spin Hamiltonian, as described in the next section.

## Spin Model and Magnetic Coupling

The FM, AFM1, AFM2, and AFM3 solutions correspond to different ordering of spins (see Figure 2) that, as detailed in the next section, correspond to estimated magnetic moments—obtained by numerical integration of electron spin densities projected in the atomic spheres—which are strongly localized in the Ti atoms. This suggests choosing the classical Heisenberg spin model Hamiltonian to rationalize the present findings. In principle, this Hamiltonian contains the two-body terms which are usually the dominating ones.^66^ Most often, only nearest neighbor terms are included. Here, to reach a more detailed description, up to three different types of two body terms are included which depend on the Ti–Ti distances in the structure. The explicit form of the Heisenberg Hamiltonian is provided in eq 1 below
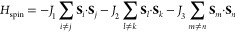
1where *J*_1_ stands for the interlayer, between the top and bottom layers, exchange coupling interaction between nearest neighbors (NN), *J*_2_ and *J*_3_ stand for the intralayer NN, and interlayer next-NN couplings, respectively. The magnetic interaction paths are schematically shown in Figure 1. All calculations lead to the same picture in which the lowest energy solutions show a spin density localized at the Ti atoms, consistent with one unpaired electron per magnetic center, *S* = 1/2. This is at variance of previous work^48^ where *S* = 1 was assumed solely on the basis of a d^2^ electronic configuration on Ti atoms with two electrons locally coupled to a triplet state, as in a Ti^2+^ cation or as in a neutral Ti atom. In the first case, one assumes that Ti_2_C is a fully ionic system with net charges matching the formal oxidation numbers; that is, Ti^2+^ and C^4–^. However, it is known that large formal charges are not physically meaningful because the fully ionic picture of the system breaks down. This is the case of titanium oxides where Ti atoms in TiO and Ti_2_O_3_ have quantum chemically calculated net charges close to +2 and +3, but this does not hold for TiO_2_ where the predicted charge is much lower that the formal one.^71^ Hence, the choice of either a fully ionic picture for Ti_2_C or of a neutral Ti atom is not consistent with the analysis of the results of the electronic structure as is discussed in the next section, indicating a partial charge transfer from Ti to C atoms as found in other previous studies.^49,72^ The picture resulting from the present calculations corresponds to a mixture character between metallic, covalent, and ionic bonding with one unpaired electron per Ti atom, consistent with a Ti^+^ center. This mixing of ionic and covalent character is also found in three-dimensional bulk carbides.^73^ Thus, the resulting electronic structure is consistent with Ti^+^ centers in a local 4s^2^3d^1^ electronic configuration with a closed-shell metallic sp band with contributions of Ti (4s) and C (2p), implying a zero gap, and a localized spin-polarized 3d band with ∼1 electron per Ti center.

Noting that the diagonal elements of the matrix representation of the Heisenberg Hamiltonian for a given solution match the total energy of the simplified Ising Hamiltonian^66^ involving the *z*-component of the spin operators only, one can immediately derive the expectation energy per formula-unit of the four ordered spin states (FM, AFM1, AFM2, and AFM3) as in eqs 2–5
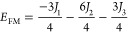
2
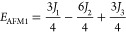
3
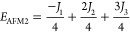
4
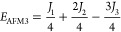
5

The different *J*_1_, *J*_2_, and *J*_3_ magnetic couplings between Ti atoms can be extracted by mapping the calculated total DFT energies of Ti_2_C per formula-unit of the different broken symmetry magnetic solutions with different spin orderings (FM, AFM1, AFM2, and AFM3) to those of the proposed Heisenberg model in eqs 2–5. Note that the use of a broken symmetry solution is dictated by the choice of a single Slater determinant in periodic calculations, and it is also intrinsic to the use of the Kohn–Sham implementation of DFT.^66,74^

## Results and Discussion

For the Ti_2_C MXene, the lattice parameters and atomic positions have been obtained employing the *p*(1 × 1) unit cell, see Figure 1 left panel, and using the optimal setup described earlier and the PBE density functional. The structural optimization has been carried out without and with spin polarization, leading to the NM and FM geometries, respectively. Next, single-point energy calculations were carried out with hybrid PBE0 and HSE06 density functionals at the NM and FM PBE geometries to examine the influence of the functional on the energy difference between these two solutions and to be able to definitively determine whether the ground state of the Ti_2_C is magnetic or not. Finally, to obtain the most stable magnetic solution, the *p*(1 × 1) unit cell is expanded to a *p*(2 × 1) supercell, see Figure 1, right panel, and the total energy of the different magnetic solutions, namely FM, AFM1, AFM2, and AFM3, have been obtained at the same structure.

In the literature, the PBE non-spin-polarized (NM) Ti_2_C structure has been reported to have metal–carbon atom (M–C) distances of 2.10 Å^48,56,75^ and a lattice constant (*a*_0_) of 3.03 ± 0.01 Å.^46,48,54,56,75−77^ These previous results are in good agreement with the present ones, as (2.10 and 3.04 Å) listed in Table 1. The small differences can be safely attributed to the differences in the computational setup which here is very tight. Likewise, the PBE spin-polarized (FM) structure for the pristine Ti_2_C MXene has been considered in the literature, and M–C distances of 2.10^56,57^ and a lattice constant of 3.07 ± 0.02 Å were reported.^45,46,56,75,78^ The results from these previous studies are also in good agreement with the values of 2.10 and 3.09 Å collected in Table 1. The effect of spin polarization in the final structure is not large but noticeable, strongly suggesting that the spin-polarized (FM) structure is the one to be used to compute the corresponding energy differences. While the difference in the relative energies for the NM and FM solutions is significant, the difference in structural parameters between different magnetic solutions is not, 0.05 Å. In fact, for the AFM1 solution, a PBE lattice parameter of 3.06 Å has been reported,^54^ which is close to the present value of 3.09 Å for the FM solution and is in excellent agreement with the present value of 3.06 Å here predicted for this AFM1 solution. From these results, one can conclude that using the lattice parameter from one of the different magnetic solutions, being at most 0.02 Å, will have a minor effect of the relevant energy differences.

**Table 1:** Distance between Metal and Carbon Atom, *d*_M–C_, and Lattice Constant, *a*_0_, Both in Å, Obtained for Ti_2_C *p*(1 × 1) Unit Cell Using PBE Functional and Considering Either the NM or the FM Solutions^a^

solution	*d*_M–C_	*a*_0_	Δ*E*_FM–NM_^PBE^	Δ*E*_FM–NM_^PBE0^	Δ*E*_FM–NM_^HSE06^
NM	2.10	3.04	–86	–450	–376
FM	2.10	3.09	–135	–488	–420

a For each optimized structure (FM and NM), the energy difference between the FM and NM solution, Δ*E* in meV, predicted by the PBE, PBE0, and HSE06 functionals is reported. Negative values indicate that the FM configuration is more stable than the NM one, taken as zero.

Regarding the nature of the Ti_2_C electronic ground state, NM solution is significantly less stable than the FM one in the *p*(1 × 1) unit cell, which is consistently found with the three studied density functionals, see Table 1. Consequently, one can firmly state that Ti_2_C has a magnetic electronic ground state. Not surprisingly, the energy differences between the NM and FM solutions (Δ*E*) at the same crystal structure are affected by the density functional by differently stabilizing the FM configuration with respect to NM one. Data in Table 1 show that for Ti_2_C structure optimized with PBE for the FM solution, the energy of the FM state relative to the NM solution is −135, −488, and −420 meV for PBE, PBE0, and HSE06, respectively. The PBE0 functional tends to stabilize more the FM with respect to the NM solution in comparison with the PBE and HSE06 functionals at the same structure, which is in the line of previous findings.^66^ Finally, we note that, for a given functional, the Δ*E* value calculated with the NM or FM crystal structure differs in less than 50 meV, clearly indicating that Δ*E* is almost independent of whether the Ti_2_C crystal structure has been optimized for the NM or FM solution. This is because spin polarization has little effect on the final optimized structure, the difference in the PBE-predicted Ti_2_C lattice constant using the NM or FM solution is ∼0.05 Å only.

Once the magnetic nature of Ti_2_C MXene electronic ground state has been established, the next step is to explore the relative energy of other possible magnetic solution. In particular, we considered the FM, AFM1, AFM2, and AFM3 spin orders, and it has been obtained the corresponding total energy for a *p*(2 × 1) supercell for these configurations. Table 2 summarizes the energy differences between each possible AFM configuration with respect to the FM as computed for a *p*(2 × 1) Ti_2_C supercell with the atomic structure optimized for the FM solution. This is justified by the small effect of the chosen solution on the resulting structure, as discussed above. The criterion to consider a given solution more stable than another is to have a significative energy difference, here taken as larger than 1 meV. For the different explored magnetic solutions, we analyze the atomic magnetic moment per Ti atom, estimated from the electronic spin density integrated in a given volume as defined in VASP. The solution is considered magnetic if the calculated spin density is larger than 0.1 unpaired electrons per Ti in absolute value. Results in Table 2 show that, for all density functionals, the electron ground state is AFM1 with the AFM2 and AFM3 solutions lying in energy above the FM solution. This is because AFM1 corresponds to the AFM coupling of the two FM metallic layers. A similar conclusion was reached by Lv et al.^48^ and by Akgenc et al.*,*^79^ both using PBE and PBE + *U* approaches, although a warning is needed regarding the choice of the *U* parameter. These authors used *U* = 4 eV, which is a common value for oxides,^80−82^ but it is not at all clear that it will be appropriate for MXenes. However, the relative stability of the different solutions varies with the density functional. Also, correlation in C atoms may play a significant role, and the effect of *U* on these atoms should be explored. This behavior is well-known^66^ and could be one of the reasons of the non-consensus found in the literature. Here, the important conclusion is that all methods and all previous work in the literature, that explored solutions other than the FM one, consistently predict a magnetic electronic ground state involving the AFM coupling of ferromagnetically coupled magnetic layers. We already mentioned that the spin model used by Lv et al.^48^ is not consistent with the nature of the chemical bond emerging from the density functional calculations. The work by Akgenc et al.^79^ deserves some additional discussion because of the inherent limitations to the classical Heisenberg Hamiltonian used and of some additional approximations. The conclusions from these authors are qualitatively correct, and they properly predict the AFM1 nature of the ground state. This approach can also be used to predict the thermodynamical properties.^83^ However, it does not follow from a rigorous quantum mechanical description. For instance, the particular form of the model Hamiltonian they use, which involves magnetic moments rather than spin operators, as in the quantum mechanical form of the Heisenberg Hamiltonian. In principle, this should not be a problem because both spin densities and magnetic moments on Ti atoms are related. The problem comes from the application of the broken symmetry approach and the use of the mapping procedure where they come out with a set of equations where energy differences between different magnetic solutions are inconsistently related to the magnitude of the magnetic moment. Next, these authors choose the magnitude of the magnetic moment as the spin density on Ti atoms provided by the GGA + *U* functional which, in turn, is roughly estimated from the integration in a sphere of arbitrary radius for just one of the magnetic solutions. In spite of its possible application to predict thermodynamic properties, such a classical model Hamiltonian cannot properly describe the energy spectrum of the spin states of discrete systems and, contrarily to what is predicted from the classical Heisenberg spin Hamiltonian, will fail to reproduce the correct splitting of the spin states and, consequently, application to periodic systems within the broken symmetry approach is not physically correct. We remind the reader that the Heisenberg Hamiltonian is a spin model that can be applied to systems with localized spins and that these must fulfill the rules of quantum mechanics for spin operators. For instance, for two particles of spin *S* = 1/2, the Heisenberg spin Hamiltonian leads to singlet and triplet states, whereas for two particles with spin *S* = 1, the spin states are singlet, triplet, and quintuplet states. A model Hamiltonian with *S* values that are not integer or half integer is not physically grounded. Finally, it is worth pointing out that the equations relating the energy of the different magnetic solutions with the set of magnetic coupling constants reported by Akgenc et al.^79^ are inconsistent with the relations reported in eqs 2–5. Apparently, the authors did not take into account the periodic structure of the supercells used since they ignored some of the equivalent interactions between magnetic centers sitting in neighboring cells, which are required in the mapping procedure for periodic systems.^84^ For more details, the reader is referred to the review paper by Moreira and Illas^66^ and the monography by some of the authors.^85^

**Table 2:** Energy of AFM1, AFM2, and AFM3 Configurations Relative to the FM One for *p*(2 × 1) Ti_2_C, Δ*E* in meV, All Computed with the PBE-Optimized FM Structure as Predicted by PBE, PBE0, and HSE06 Functionals^a^

Δ*E*	PBE	PBE0	HSE06
AFM1-FM	–45	–187	–149
AFM2-FM	172	313	293
AFM3-FM	160	336	301

a Negative values indicate that the AFM_*i*_ (*i* = 1–3) configuration is more stable than the FM one and positive ones, vice versa.

Table 3 summarizes the total net spin densities per supercell, the atomic spin density on metal and carbon atoms for FM configuration, and the atomic metal spin densities of the three AFM configurations for each density functional. For all solutions, the spin densities per Ti atom are larger than 0.1 unpaired electrons per Ti for all three functionals as expected for a robust spin-polarized solution. In addition, the total net spin density for the *p*(1 × 1) unit cell with PBE, PBE0, and HSE06 is 1.92, 1.85, and 1.92 unpaired electrons per unit cell, respectively, and is in good agreement with previous calculations.^38,45,46,48,52,55^ Likewise, for all density functionals, the atomic spin densities from the FM solution for Ti_2_C in Table 3 match the reported ones,^48^ except for results reported in ref (46) which are about 0.4 unpaired electrons per atom larger than the present ones in Table 3; this difference is attributed to the different computational setup which affects the space partition of the spin density. For the AFM1 solution, the atomic spin densities predicted by the PBE and hybrid functionals, see Table 3, are in qualitative agreement with those reported in previous studies,^42,48,49^ although with some discrepancies in the numerical values which are attributed to the lack of a common, tight enough, numerical setup in the literature. Interestingly, for the AFM2 and AFM3, the Ti spin densities calculated with all density functionals, see Table 3, are very close to each other and are in good agreement with the ones reported in the literature considering AFM solutions.^48^ Here one must point out that, for the AFM2 and AFM3 solutions, the estimated magnetic moment is significantly smaller than that for the AFM1. This is due to the large energy penalty necessary to invert spins in each of the metal layers, evidencing a clear competition between magnetic interactions and chemical bonding leading, in these solutions, to a much smaller spin density. Finally, note that, in all cases and for all functionals, the polarization of spin density is mainly due to d-electrons of the Ti atoms, consistent with one unpaired electron per Ti, and all values assigned to C atoms in Table 3 are residual.

**Table 3:** Total Net Spin Densities of the *p*(2 × 1) Ti_2_C Supercell, *M*_Tot_^FM^, Given in Unpaired Electrons per Supercell, Atomic Spin Densities of Ti and C Atoms of FM Configuration, and the Atomic Spin Densities of Ti Atoms in the Different Solution, *M*_Atom_^Solution^, Given in Unpaired Electrons per Atom, in Absolute Value for the AFM Ones, as Predicted by PBE, PBE0, and HSE06 Functionals

	PBE	PBE0	HSE06
*M*_Tot_^FM^	3.85	3.85	3.85
*M*_Ti_^FM^	0.54	0.55	0.55
*M*_C_^FM^	–0.04	–0.07	–0.07
*M*_Ti_^AFM1^	0.56	0.72	0.72
*M*_Ti_^AFM2^	0.21	0.33	0.32
*M*_Ti_^AFM3^	0.24	0.35	0.34

In order to accurately analyze the magnetic nature of the ground state, the magnetic coupling parameters, *J*_1_, *J*_2_, and *J*_3_, of the spin Heisenberg Hamiltonian in eq 1, are calculated from the difference between the total energy of FM and AFM states per formula-unit based on eqs 2–5. To avoid any bias, these parameters are extracted from the energy differences per formula unit corresponding to each of the three density functionals as explained above with values from the different functional providing accurate and robust energy range. The calculated *J*_1_, *J*_2_, and *J*_3_ are listed in Table 4. *J*_1_ is calculated to be within the range −13 and −41 meV; *J*_2_, within the 47 and 105 meV range and *J*_3_, within the range −1 and −21 meV. As expected, the magnetic couplings thus derived strongly depend on the employed functional,^66^ but the overall picture remains. Thus, no matter the functional used, *J*_2_ is by far the dominant term, but *J*_1_ is rather large but opposite sign, whereas *J*_3_ is the smallest but noticeable term. Overall, magnetic interactions cannot be reduced to a nearest-neighbor problem. The largest *J* values are those predicted by the PBE0 hybrid density functional, in the same way that happens with the Δ*E*(FM–NM) and Δ*E*(AFM_*i*_–FM; *i* = 1–3) values discussed above. The fact that the absolute value of *J*_2_ is larger than the absolute values of *J*_1_ and *J*_3_ implies that the intralayer magnetic interactions between Ti atoms are stronger than the ones involving interlayer Ti atoms. Positive/negative values of *J* indicate the preference for FM/AFM coupling, and *J*_1_ ≪ 0 and *J*_3_ < 0. All in all, this is consistent with a Ti_2_C ground state involving the AFM coupling between ferromagnetically coupled layers.

**Table 4:** Spin Exchange Parameters, *J*_1_, *J*_2_, and *J*_3_, Given in meV, as Obtained from DFT Calculations for the Three Studied Functionals Using the Equations Derived from the Mapping of the Broken Symmetry Solutions to the Heisenberg Spin Model Hamiltonian, See Eqs 2–5

	PBE	PBE0	HSE06
***J***_1_	–13.995	–40.999	–35.296
***J***_2_	47.074	104.608	92.810
***J***_3_	–0.919	–21.516	–14.292

To finalize the discussion, we make use of the mean field approximation to roughly estimate the Néel temperature, *T*_N_, using the predicted magnetic coupling constant range. This represents a quite drastic approximation, implying that the predicted values must be taken with extreme care and just to provide a realistic temperature range rather than a precise value even if this is obtained from accurate values of the magnetic coupling constants, as predicted from calculations using hybrid functionals. Following Pajda et al.,^86^*T*_N_ can be roughly estimated for a 3D magnetic system using eq 6
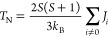
6where *S* is the total spin quantum number per magnetic center, *k*_B_ is the Boltzmann constant, and *J*_i_ correspond to the *J*_1_, *J*_2_, and *J*_3_ already discussed. From the magnetic moments estimated from spin densities in Table 3 already discussed, choosing *S* = 1/2 represents a consistent choice, rather than *S* = 1 used previously that would correspond to two unpaired electrons per Ti atom locally couplet to a triplet state.^48^ The *T*_N_ thus calculated is in the 187–251 K range, or 220 ± 30 K, where the lower bound is obtained from the PBE *J*_1_, *J*_2_, and *J*_3_ values, and the upper bound corresponds to the values predicted by the hybrid HSE06 functional with an intermediate value of 244.24 K predicted by the PBE0 hybrid functional. It is worth pointing out that the range for *T*_N_ involves temperatures high enough to make the Ti_2_C MXene well suited for practical applications in spintronics. Note also that previous work by Lv et al.^48^ concluded that AFM1 is the ground state for Ti_2_C, which is in full agreement with the present findings. However, also using eq 6, these authors reported a much lower value of *T*_N_ (50 K). There are two reasons to explain this difference: the first one is on the spin model since these authors *S* = 1, which is not consistent with the analysis of spin density, and the second one is the use the PBE + *U* functional with *U* = 4 eV which, in the view of the results obtained with the more accurate HSE06 and PBE0 hybrid functionals, does not seem to be a proper choice for this system.

## Conclusions

An accurate and systematic study has been presented aimed at unambiguously determining the nature of the electronic ground state of the Ti_2_C MXene. This has been achieved using different density functionals and a very tight setup to providing numerically converged results up to 1 meV. Regarding the crystal structure, the lattice constants and M–C distances predicted here for non-spin-polarized and different spin-polarized solutions obtained using the PBE functional are in good agreement with each other with the calculated lattice parameter differing at most by 0.02 Å and also agree with previously reported ones. In fact, the present results prove that the effect of the spin polarization on the structural parameters is negligible but obviously affects the total energy stability of the different magnetic solutions.

The PBE, PBE0, and HSE06 density functionals consistently predict that the Ti_2_C MXene has a magnetic ground state and is in agreement with the available literature. Moreover, for Ti_2_C, the most stable magnetic solution is the AFM1 using the three studied functionals. A result, which was reported by several previous articles^42,48,53−57^ whereas some other, sometimes incompletely, just explored the FM solution. As expected, the density functional has a large effect on the relative stability of the AFM, FM, and NM solutions. For the same crystal structure, the hybrid PBE0 density functional tends to stabilize more the FM state over the NM solution than the PBE and HSE06 functionals. In addition, the PBE functional underestimates the relative stability of the electronic ground state of Ti_2_C because of an excessive delocalization of the electron density although it correctly predicts the ground state and provides qualitatively correct estimations of atomic spin densities. All in all, all methods lead to the same conclusion, namely, that the Ti_2_C ground state involves the AFM coupling of ferromagnetically coupled layers.

The present spin model involving one unpaired electron per Ti is consistent with the nature of the chemical bond arising from the calculations indicating a strong ionic component and differs from the previous work of Lv et al.,^48^ which assumed two unpaired electrons, locally coupled to triplet, as in a completely ionic material or in the isolated Ti atom. An estimation of the magnitude of the relevant magnetic coupling constants extracted from appropriate energy differences per formula unit is also reported. Even more, using three different density functionals makes it possible to estimate a range for the magnitude of each of the explored parameters. In all cases, the intralayer FM coupling, *J*_2_, is the dominant interaction in the system but the other two couplings are noticeable and of opposite sign. This implies that these terms cannot be negligible and that the spin model cannot be reduced to include nearest-neighbor interactions only. Finally, a very rough estimate of the order of magnitude of the Néel temperature for this complex 2D magnetic system is provided that is 220 ± 30 K involving values high enough to suggest that this material can be used in practical applications.

## Supporting Information Available

VASP input files for the calculation of the different solutions using the *p*(2 × 1) supercell (PDF)
