# Supplementary material for: Theoretical Analysis of Magnetic Coupling in the Ti2C Bare MXene
Source: J Phys Chem C Nanomater Interfaces. 2023 Feb 14;127(7):3706–14. doi: 10.1021/acs.jpcc.2c07609 (PMC9969871; doi:10.1021/acs.jpcc.2c07609)
Supplement: Supplementary file 1 — jp2c07609_si_001.pdf [file jp2c07609_si_001.pdf]

A Theoretical Analysis of Magnetic Coupling in the Ti_2C Bare MXene

Néstor García-Romeral, Ángel Morales-García, Francesc Viñes, Ibério de P. R Moreira, Francesc Illas*

Departament de Ciència de Materials i Química Física & Institut de Química Teòrica i Computacional (IQTCUB), Universitat de Barcelona, c/ Martí i Franquès 1-11, 08028 Barcelona, Spain

*Corresponding author: francesc.illas@ub.edu

Input files used to compute the energy and electronic structure of each electronic state (NM, FM, AFM1, AFM2, and AFM3) over the *p*(1×1) and *p*(2×1) FM structures optimized with PBE.

POSCAR for *p*(1×1) FM structure:

```
Ti2C
  1.00000000000000
    3.0855893780612158    0.0000000000000000    0.0000000000000000
    1.5427946890306079    2.6712992808823635   -0.0001996281757864
    0.0000000000000000   -0.0014580244524128   18.6208052430075135
  Ti   C
    2     1
Direct
  0.3333477472655630  0.3333045054588766  0.3066513547538818
  0.6666522527344370  0.6666954945411305  0.4262591935161240
  0.0000000000000000  0.0000000000000000  0.3664552741400016
```

POSCAR for *p*(2×1) FM structure:

```
Ti2C
   1.00000000000000
     6.1722364426000000    0.0000000000000000    0.0000000000000000
     1.5414232899000000    2.6716893598000002    0.0000000000000000
    -0.0064958858000000    0.0031550110000000   18.6208052430075135
   Ti   C
     4     2
Direct
  0.1668858298117470  0.3333361288881704  0.2763880102302565
  0.6668816722686088  0.3333617888196656  0.2763907468466016
  0.3336098161497247  0.6666922975989706  0.3960646236160770
  0.8335860936271260  0.6667178677704513  0.3960638137692527
  0.0002176023535156  0.0000163888700442  0.3362290116508859
```

KPOINTS:

K-Points

0

Monkhorst Pack

13 13 1

0 0 0

INCAR for $p(1\times1)$ FM structure and NM electronic state:

```
IBRION =  -1
POTIM  =  0.5
EDIFFG =  -0.01
NSW    =  0

EDIFF  =  1E-06
ISPIN  =  1
LORBIT = 11
ISMEAR =  1
SIGMA  =  0.01

LREAL  =  .TRUE.

NWRITE =  2
NELM   =  101
NELMIN =  2
ENCUT  =  700
IALGO  =  58

ISTART =  0
INIWAV =  1

GGA    =  PE
```

INCAR for $p(1\times1)$ and $p(2\times1)$ FM structure and FM electronic state:

```
IBRION =  -1
POTIM  =  0.5
EDIFFG =  -0.01
NSW    =  0

EDIFF  =  1E-06
ISPIN  =  2
LORBIT =  11
ISMEAR =  1
SIGMA  =  0.01

LREAL  =  .TRUE.

NWRITE =  2
NELM   =  101
NELMIN =  2
ENCUT  =  700
IALGO  =  58

ISTART =  0
INIWAV =  1

GGA    =  PE
```

INCAR for *p*(2×1) FM structure and AFM1 electronic state:

```
IBRION =  -1
POTIM  =  0.5
EDIFFG =  -0.01
NSW    =  0

EDIFF  =  1E-06
ISPIN  =  2
MAGMOM = -1.0 -1.0 1.0 1.0 2*0.0
NUPDOWN=  0.0
LORBIT =  11
ISMEAR =  1
SIGMA  =  0.01

LREAL  =  .TRUE.

NWRITE =  2
NELM   =  101
NELMIN =  2
ENCUT  =  700
IALGO  =  58

ISTART =  0
INIWAV =  1

GGA    =  PE
```

INCAR for *p*(2×1) FM structure and AFM2 electronic state:

```
IBRION =  -1
POTIM  =  0.5
EDIFFG =  -0.01
NSW    =  0

EDIFF  =  1E-06
ISPIN  =  2
MAGMOM = -1.0 1.0 -1.0 1.0 2*0.0
NUPDOWN=  0.0
LORBIT =  11
ISMEAR =  1
SIGMA  =  0.01

LREAL  =  .TRUE.

NWRITE =  2
NELM   =  101
NELMIN =  2
ENCUT  =  700
IALGO  =  58

ISTART =  0
INIWAV =  1

GGA    =  PE
```

INCAR for *p*(2×1) FM structure and AFM3 electronic state:

```
IBRION =  -1
POTIM  =  0.5
EDIFFG =  -0.01
NSW    =  0

EDIFF  =  1E-06
ISPIN  =  2
MAGMOM =  1.0 -1.0 -1.0 1.0 2*0.0
NUPDOWN=  0.0
LORBIT =  11
ISMEAR =  1
SIGMA  =  0.01

LREAL  =  .TRUE.

NWRITE =  2
NELM   =  101
NELMIN =  2
ENCUT  =  700
IALGO  =  58

ISTART =  0
INIWAV =  1

GGA    =  PE
```
